# Dr. Boys, on the Medical Officers of the Navy

**Published:** 1807-03

**Authors:** Edward Boys

**Affiliations:** Physician to the Royal Navy. Boyne Hospital, Haslar


### *To the Editors of the Medical and Physical Journal.*

GENTLEMEN,

It had been so usual for me to experience gratification in the perusal of the Medical and Physical Journal, that it was with infinite regret I witnessed in the latter Numbers of it, the introduction of a subject which should be accounted totally foreign to the nature and essence of the work; I allude to a very grave attack which has been made on the character of a particular class of gentlemen, the Surgeons of the Royal Navy, and who now, in consequence, have have a right to claim from you, a place in this Journal, for any thing which they may have to say on the subject, and thus a considerable portion of a valuable work may be occupied by unusual matter, by a controversy that has not been looked for by the public, and that cannot, I suppose, be acceptable to it. I am not wrong in asserting that the Surgeons of the Navy possess both spirit and abilities to meet any accusation, although I am not so sure that they may think it either proper or necessary to notice what has been advanced against them.

There are about six hundred officers on the list of Navy Surgeons; and at a time when two-thirds of these gentlemen are employed in the defence of their country, and in situations where the Medical and Physical Journal cannot reach them, was it to be expected that this publication should be the means, through which they are accused to the world, of "*not interesting themselves in their duty?*" and "of *their want of ability to perform it?*" and that it should mention farther of them, that "*these defects generally apply to the medical department of the navy?*" These are, doubtless, pointed assertions, of much importance, and ought only to have arisen from a source in possession of great information in that particular branch of the public service to which they are applied.

I have two motives for writing this letter; to give my testimony, such as it may be, to the merits of the surgeons of the navy, and to complain to you, Gentlemen, that I think unfavourably of your having permitted a periodical publication of repute, to be the vehicle through which the character of any body of men has been detracted from.*

When I shall say of myself, that after preliminary service, I was one of four officers who were selected from the list of Navy Surgeons, in January 1798, to be sent to France, for the purpose of seeing British prisoners of war taken care of in that country; that I was for more than five years the surgeon in charge of the whole duty of a

 considerable

* The Editors insert this rebuke with great pleasure, because their conduct has given the Medical Officers of the Navy, and Dr. B. in particular, an opportunity of repelling any unfounded or illliberal censures passed on so important and respectable a body of Medical Practitioners. But if, on the other hand, any Surgeons in the Navy are found who merit such censures, this publication will stimulate them to acquire the knowledge necessary for their situation. If any Gentleman, who *gives his Name* to the public, passes a rash or unmerited censure on an individual or body of men, the injury recoils on himself. The public will decide rightly.

considerable Naval Hospital, in the late and present wars; and that I have been now almost three years one of the physicians of this establishment, during which periods some thousand cases, physical and surgical, have been placed under my care, by different surgeons of the navy, from the ships of his Majesty's fleet: I trust my voice, on this occasion, may be entitled to some consideration; and I do therefore beg leave to assure you, gentlemen, and your readers, that the observations which I have had opportunities for making during my public services, have led me to form opinions on the assiduity and abilities of the Navy Surgeons *in general,* (whether I have witnessed them employed in the fleet or in the hospitals) quite in opposition to the assertions of Mr. D.; and I do the Surgeons of the Navy only justice in stating, that I have often seen them, by perseverance and attention, surmount difficulties peculiarly attendant on their situations.

Among any body of men, there are always some to be found, who do not possess equal abilities with others, and particular circumstances of neglect and want of skill, when they may be brought forward, should be directed to apply in their proper places; but, on this account, has it been either reasonable or decorous to cast stigmas on a whole corps? on men, whose skill and humanity have been acknowledged, on many memorable occasions, to have solaced heroes bleeding for their country? Their superiors have not been backward in remembering, that the Surgeons of the Navy, partook of the honours of St. Vincent's, of Camperdown, Aboukir, and Trafalgar, or that they shared the dangers and the labours consequent to those and many other victories.

I will now, Gentlemen, beg leave to say adieu to you, with these observations, that had the medical cases in dispute, alone been touched on in the Medical and Physical Journal, sufficient abilities have been evinced by all parties, to have promised to your readers useful information from the controversy; but it surely was your duty to have guarded against the introduction of highly objectionable matter, although it was attempted to be thrust on the public eye, under the cloak of "Remarks on a Case of diseased Bladder" and "of Gastritis," or "on Pneumonia."

I am, &c.

EDWARD BOYS,
Physician to the Royal Navy.

*Boyne Hospital, Haslar,*

*Feb.* 9, 1807.

